# Predictors of emergency room visits or acute hospital admissions prior to death among hospice palliative care clients in Ontario: a retrospective cohort study

**DOI:** 10.1186/1472-684X-13-35

**Published:** 2014-07-11

**Authors:** Lialoma Salam-White, John P Hirdes, Jeffrey W Poss, Jane Blums

**Affiliations:** 1 School of Public Health and Health Systems, University of Waterloo, 200 University Avenue West, Ontario N2L 3G1, Canada; 2 Hamilton Niagara Haldimand Brant Community Care Access Centre (HNHB CCAC), 211 Prichard Road, Unit 1, Hamilton, Ontario L8J 0G5, Canada

**Keywords:** Hospice palliative care, Emergency room visits, Acute hospital admissions, InterRAI PC

## Abstract

**Background:**

Hospice palliative care (HPC) is a philosophy of care that aims to relieve suffering and improve the quality of life for clients with life-threatening illnesses or end of life issues. The goals of HPC are not only to ameliorate clients’ symptoms but also to reduce unneeded or unwanted medical interventions such as emergency room visits or hospitalizations (ERVH). Hospitals are considered a setting ill-prepared for end of life issues; therefore, use of such acute care services has to be considered an indicator of poor quality end of life care. This study examines predictors of ERVH prior to death among HPC home care clients.

**Methods:**

A retrospective cohort study of a sample of 764 HPC home care clients who received services from a community care access centre (CCAC) in southern Ontario, Canada. All clients were assessed using the Resident Assessment Instrument for Palliative Care (interRAI PC) as part of normal clinical practice between April 2008 and July 2010. The Andersen-Newman framework for health service utilization was used as a conceptual model for the basis of this study. Logistic regression and Cox regression analyses were carried out to identify predictors of ERVH.

**Results:**

Half of the HPC clients had at least one or more ERVH (n = 399, 52.2%). Wish to die at home (OR = 0.54) and advanced care directives (OR = 0.39) were protective against ERVH. Unstable health (OR = 0.70) was also associated with reduced probability, while infections such as prior urinary tract infections (OR = 2.54) increased the likelihood of ERVH. Clients with increased use of formal services had reduced probability of ERVH (OR = 0.55).

**Conclusions:**

Findings of this study suggest that predisposing characteristics are nearly as important as need variables in determining ERVH among HPC clients, which challenges the assumption that need variables are the most important determinants of ERVH. Ongoing assessment of HPC clients is essential in reducing ERVH, as reassessments at specified intervals will allow care and service plans to be adjusted with clients’ changing health needs and end of life preferences.

## Background

Hospice palliative care (HPC) is a client and family centred philosophy of care that aims to improve the quality of life of individuals with life-threatening illnesses or end of life issues [1]. The goals of HPC are not only to improve clients’ symptoms, but also to reduce disruptions and distress caused by unneeded or unwanted medical interventions, including avoidable emergency room visits or hospitalizations (ERVH) [2]. The excessive use of acute care services is considered by some to be an indicator of poor quality end of life care [3-5].

HPC is used interchangeably or in combination with palliative care, hospice care and end of life care. HPC is a broad term that includes hospice care (which focuses on relieving suffering), and palliative care (which aims to improve quality of life and other aspects of end of life care) [6]. HPC is a descriptor that is used by the Canadian Hospice Palliative Care Association and in some literature [7-10] because it can be provided in a variety of settings including a client’s home, an inpatient hospice facility, a specialized unit in a hospital, and a continuing care facility.

ERVH among HPC clients living at home has been the subject of limited research in Canada, with only two studies in the past few years. Lawson and colleagues (2008) reported that 27% (5,803 out of 21, 323) Of Nova Scotian palliative clients in a home care program, outpatient clinic, and inpatient unit made at least one visit to the emergency room in the last 6 months of life. Over half of these visits resulted in hospital admissions. The primary reasons for emergency room visits included pain and shortness of breath [11]. Similar reasons for emergency room visits have been documented in other countries [12-14]. Brink and colleagues (2011) reported that 35% (33 out of 93) of Ontario HPC clients visited an emergency room within 45 days of being enrolled in a HPC program and that the main risk factors for emergency room visits included weight loss and previous hospitalization [15]. These two variables increased the likelihood of emergency room use, while greater cognition impairments reduced the likelihood of emergency department use [15].

Some hospitals may be ill-prepared for end of life issues, but there is variability in practice patterns because some have specialized units for palliative care while others set aside a certain number of beds [12,16,17]. Nonetheless, almost 60% of deaths occur in hospital settings [18]. ERVH may not be appropriate for HPC clients since it has been shown that, in some cases, hospital based clinicians may have limited relationships with their patients and may not have knowledge of the person’s immediate illness situation, wishes, or values to guide decisions around end of life care [19].

Determining which variables predict ERVH, by community-dwelling HPC clients, could help to identify clients who could benefit from a care plan to prevent these visits. This study examines client characteristics that may predict ERVH among HPC home care clients in Ontario, Canada. The characteristics are defined within the behavioural model for health service utilization as described by Andersen and Newman. The model describes individual determinants of health service utilization according to predisposing, enabling, and illness (or need for care) variables [20]. Predisposing characteristics include demographic, social-structure, and attitudes and beliefs related to the use of health services. Enabling factors include family structure and community factors which make health services available, accessible, and affordable. Illness variables include client’s perceived illness and the professionals’ evaluation of the client’s illness. Many studies have determined predisposing characteristics as having a moderate effect, enabling factors as being the most distal cause of service use, while need-based variables as the most proximate [21-32]. Although this model has been used among the elderly population, there are not many cases where it has been used among a HPC population.

## Methods

### Sample

This study was designed as a retrospective cohort study of HPC home care clients who received services from a community care access centre (CCAC) in southern Ontario, Canada. The sample included clients assessed with interRAI Palliative Care (PC) instrument between April 2008 and July 2010 and who died within one year of the assessment. All clients were assessed at home and had at least a one-day period where an ERVH could have occurred.

In Ontario, there are 14 CCACs that are funded and legislated by the Ministry of Health and Long-Term Care (MOHLTC). Among their responsibilities is the delivery of home care services to individuals, enabling them to remain at home for as long as possible and delaying or preventing admission to hospitals or long-term care homes [33]. There are no age restrictions or charges for services provided to Canadian citizens and permanent residents. Individuals requiring services can refer themselves for services, or they can be referred through a family member, caregiver, friend, physician, or other health care professionals [33]. Care coordinators, who are regulated health professionals, are responsible for coordinating service delivery. Care coordinators assess client needs, determine eligibility for services, and identify the nature, intensity and duration of services required. The assessment ensures that the right services are provided to clients at the right time and reassessments can be done on a needed basis. Some of the services provided by CCACs include nursing, personal support, physiotherapy, occupational therapy, speech-language therapy, social work, nutritional counselling, medical supplies and equipment, as well as information about and referral to other community services [33]. In addition, respite care services where clients are admitted to a nursing home for a maximum of 60 days, are available for caregivers who require temporary relief from their caregiving duties [33].

Clients are considered palliative and services such as nursing and personal support are ordered immediately based on the following criteria: clients have a life limiting or life threatening health condition, regardless of diagnosis; clients may have a prognosis of six months or less to live; or clients have pain and symptom issues related to end of life conditions [34].

### Data source

A business intelligence information system containing data on all area hospitals and the home care agency records was the source of all data used in the present study. Specifically, de-identified client level information was abstracted from the Canadian Institute for Health Information (CIHI) acute care information systems, the CCAC’s Client Health Related Information System (CHRIS), and the interRAI PC. Client records were linked to acute care service use data by person-level identifiers, available at the CCAC providing HPC services.

Acute care data gathered by CIHI in Ontario include the National Ambulatory Care Reporting System (NACRS) that contains information on emergency department visits [35] and the Discharge Abstract Database (DAD) that contains information on acute hospital admissions [36]. The CCAC’s CHRIS system includes demographic and service used information on home care clients [37], and the interRAI PC assessment system contains interRAI PC data for end of life clients receiving HPC services.

The interRAI PC assessment is used to evaluate needs, strengths, and preferences of clients in palliative care settings [38-40]. In each CCAC, case managers are trained in using the interRAI PC, as part of normal clinical practice, to assess client needs and develop a supportive care plan [38,39,41]. The assessment includes basic demographic information and covers domains including cognitive and physical functioning, mood, health conditions, and service utilization [38,40]. The interRAI PC has been shown to have good inter-rater reliability in two large scale international studies [42,43]. There are several embedded scales available within the interRAI PC. The scales used in these analyses are the Activities of Daily Living Hierarchy scale (ADLH) that assesses independent living skills [44], the Changes in Health End stage disease and Signs and Symptoms scale (CHESS) that predicts mortality and instability in health [44,45], and the Pain scale that measures frequency and severity of pain [46]. For all scales, higher scores indicate greater functional impairment and more severe symptoms.

Ethics clearance for secondary data analyses was granted from the University of Waterloo Office of Research Ethics (ORE#17640).

### Measures

Independent variables were selected based on literature reviews and palliative care clinicians’ feedback [47-50]. Most of the independent variables were divided into binary variables and categorized according to predisposing, enabling, and need variables. The variable wish to die at home had 3 options, “no”, “yes” and “unable to determine”; about half that were documented as “unable to determine” were grouped as “no”, allowing this variable to be collapsed into two groups, “yes” and “no”. An indicator of urban or rural residence was created using a zero in the second position of the client’s postal code, which was derived from the interRAI PC. Client’s cost of care, above or below the median, was derived from linked home care service data using formal services from the week prior to the interRAI PC assessment and from two weeks following the assessment. Although the interRAI PC has a section to document clients’ disease diagnoses, it does not have enough granularity for infections. Therefore, pneumonia and urinary tract infections (UTI) were retrieved from the NACRS and DAD databases. In order for the infections to remain relevant, only those resulting in a hospital visit up to one month prior to the interRAI PC assessment were included. Both infections are considered as a marker for a client’s general state because they may have been related to the impaired immune function, anatomic and functional changes accompanying the client’s terminal illness [51]. All variables included in the analyses are shown in Tables 1, 2 and 3.

**Table 1:** Predisposing characteristics: descriptive characteristics of clients with at least one emergency department visits or hospitalizations (n = 764)

**Variable**	**Sample % (n)**	**Rate of ERVH % (n)**	**p value**	**Unadjusted**	**95% Confidence interval**
				**Odds ratio**	
**Significant variables (based on p value)**					
**Wish to die at home**^**1**^					
*No*	70.5 (539)	59.7 (322)	p < .01	1	
*Yes*	29.4 (225)	34.2 (77)		0.35	0.25-0.48
**Advance directives**^**2**^					
*No*	47.8 (365)	66.6 (243)	p < .01	1	
*Yes*	52.2 (399)	39.1 (156)		0.32	0.24-0.43
**Marital status**					
*Single*	48.2 (368)	46.1 (182)	0.0006	1	
*Married*	51.8 (396)	58.7 (216)		1.65	1.24-2.20
**Age**					
*Aged <75*	60.4 (465)	56.5 (263)	0.003	1	
*Aged 75+*	31.6 (215)	45.5 (136)		0.64	0.48-0.86
**Not significant variables (based on p value)**					
**Finds guidance in religion or spirituality**^**3**^					
*No*	51.6 (370)	51.9 (186)	0.29	1	
*Yes*	48.4 (394)	54.1 (213)		1.16	0.88-1.55
**Sex**					
*Male*	52.8 (401)	53.9 (216)	0.45	1	
*Female*	47.2 (358)	50.9 (182)		0.92	0.69-1.22

*Note:* Odds ratio = 1 indicates reference group.

^1^Response obtained from client at time of assessment.

^2^Includes do not resuscitate, do not intubate, do not hospitalize, do not send to emergency department, do not tube feeding, and medication restriction.

^3^Person finds inspiration and support from religious activities, materials, and/or leaders.

**Table 2:** Enabling factors: descriptive characteristics of clients with at least one emergency department visits or hospitalizations (n = 764)

**Variable**	**Sample % (n)**	**Rate of ERVH % (n)**	**p value**	**Unadjusted**	**95% Confidence interval**
				**Odds ratio**	
**Significant variables (based on p value)**					
**Person’s median cost of care**					
*Less than or equal to $562.90*	49.9(360)	59.7(215)	0.0001	1	
*Greater than $562.90*	50.1(361)	45.4(164)		0.59	0.45-0.79
**Caregiver distress**^**1**^					
*No*	84.3 (644)	54.7 (352)	0.002	1	
*Yes*	15.7 (120)	39.2 (47)		0.53	0.36-0.79
**Not significant variables (based on p value)**					
**Access to a physician**^**2**^					
*No*	1.4 (11)	45.4 (5)	0.65	1	
*Yes*	98.6 (753)	52.3 (394)		1.32	0.40-4.35
**Rural character**					
*No*	88.2 (674)	52.1 (351)	0.99	1	
*Yes*	11.8 (90)	52.5 (47)		1.00	0.64-1.55

*Note:* Odds ratio = 1 indicates reference group.

^1^Caregiver expresses feelings of distress, anger, or depression/is unable to continue caring activities.

^2^Whether clients had a physician contact on file.

**Table 3:** Need variables: descriptive characteristics of clients with at least one emergency department visits or hospitalizations (n = 764)

**Variable**	**Sample % (n)**	**Rate of ERVH % (n)**	**p value**	**Unadjusted**	**95% Confidence interval**
				**Odds ratio**	
**Significant variables (based on p value)**					
**Believes physical function can improve**^**1**^					
*No*	77.6 (593)	48.6 (256)	p < .01	1	
*Yes*	22.4 (171)	67.8 (116)		2.31	1.61-3.31
**Bladder/bowel incontinence**					
*No*	71.9 (538)	58.5 (315)	p < .01	1	
*Yes*	28.1 (210)	36.4 (76)		0.41	0.29-0.57
**Swallowing difficulties**					
*No*	71.4 (527)	57.9 (305)	p < .01	1	
*Yes*	28.6 (211)	38.1 (80)		0.46	0.33-0.64
**Potential delirium**^**2**^					
*No*	84.2 (624)	57.0 (355)	p < .01	1	
*Yes*	15.8 (117)	30.8 (36)		0.35	0.23-0.53
**Activities of daily living Hierarchy scale**^**3**^					
*(0) Independent*	48.4 (335)	67.2 (225)	p < .01	1	
*(1,2,3,4,5,6) Dependent*	51.6 (357)	41.3 (147)		0.44	0.33-0.59
**Cognitive skills for daily decision making**^**4**^					
*(0) Intact*	78.0 (574)	58.2 (334)	p < .01	1	
*(1,2,3,4,5) Impaired*	22.0 (162)	32.7 (53)		0.36	0.25-0.52
**Chess scale**^**5**^					
*(0,1,2) Stable Health*	34.4 (239)	66.1 (158)	p < .01	1	
*(3,4,5)Unstable Health*	65.6 (455)	44.8 (204)		0.47	0.35-0.64
**Weight loss**^**6**^					
*No*	49.3 (377)	57.3 (216)	0.005	1	
*Yes*	50.6 (387)	47.3 (183)		0.67	0.50-0.89
**Pressure ulcer**					
*No*	85.5 (645)	55.2 (348)	0.01	1	
*Yes*	14.5 (109)	46.7 (45)		0.60	0.40-0.90
**Peripheral edema**					
*No*	67.1 (501)	55.2 (276)	0.03	1	
*Yes*	32.9 (246)	46.7 (115)		0.72	0.53-0.98
**Previous hospitalization (last 90 days)**					
*No hospitalization within 90 days*	32.5 (248)	58.9 (146)	0.03	1	
*31 to 90 days ago*	36.4 (278)	50.4 (140)		0.63	0.44-0.90
*In the last 30 days*	31.1 (238)	47.5 (113)		0.71	0.50-1.0
**Not significant variables (based on p value)**					
**Cancer**					
*No*	12.0 (92)	42.9 (39)	0.07	1	
*Yes*	88.0 (672)	53.4 (359)		1.49	0.96-2.31
**Dizziness**					
*No*	68.1 (505)	54.4 (274)	0.09	1	
*Yes*	31.9 (237)	47.7 (113)		0.77	0.56-1.04
**Falls in the last 90 days**					
*No*	74.4 (549)	54.6 (299)	0.10	1	
*Yes*	25.6 (189)	47.6 (90)		0.78	0.56-1.09
**Instrumental activities of Daily living**^**7**^					
*(0) Independent*	4.0 (30)	66.7 (20)	0.10	1	
*(1,2,3,4,5,6,8) Dependent*	96.0 (718)	51.2 (370)		0.62	0.34-1.15
**Diabetes**					
*No*	92.8 (709)	51.5 (365)	0.23	1	
*Yes*	7.2 (55)	60.0 (33)		1.41	0.80-2.46
**Prior urinary tract infection**					
*No*	95.5 (730)	94.74 (378)	0.26	1	
*Yes*	4.4 (34)	5.26 (21)		1.50	0.74-3.05
**Prior pneumonia**					
*No*	93.2 (712)	92.7 (370)	0.60	1	
*Yes*	6.8 (52)	7.3 (29)		1.16	0.66-2.05
**Fatigue**					
*No*	0.7 (5)	60.0 (3)	0.71	1	
*Yes*	99.3 (730)	51.7 (377)		0.66	0.33-1.34
**Dyspnea**					
*No*	30.8 (226)	53.1 (120)	0.85	1	
*Yes*	69.2 (508)	52.3 (265)		1.02	0.75-1.37
**Pain**					
*(0) Not Present*	25.9 (187)	52.9 (99)	0.96	1	
*(1,2,3) Present*	74.1 (536)	53.2 (285)		1.14	0.83-1.55

*Note:* Odds ratio = 1 indicates reference group.

^1^The client’s perception regarding his or her ability to improve in the area of physical functioning.

^2^Periodic disordered thinking or awareness (i.e. easily distracted, episodes of disorganized speech, and mental function varies over the course of the day).

^3^Assesses basic independent living skills based on items such as eating, personal hygiene, toileting, and locomotion within the home.

^4^Person’s actual performance in making everyday decisions about the tasks or activities of daily living (e.g., choosing items of clothing, knowing when to eat meals).

^5^Changes in Health, End Stage Disease, Signs and Symptoms: a scale that predicts mortality and instability in health based on items such as weight loss, shortness of breath, vomiting, dehydration, leaving food uneaten, and peripheral edema.

^6^Weight loss of 5% or more in the last 30 days, or 10% or more in the last 180 days.

^7^Self-performance in areas of function that are most commonly associated with independent living (i.e., meal preparation, ordinary housework, managing medications).

There were missing values for some of the explanatory variables from the interRAI PC because data collection during a pilot implementation was paper-based rather than computerized. Therefore, automated checks to require completeness were not possible at the time of assessment. Variables with excessive rates of missing data (60 or more) were excluded from the analyses.

### Outcomes of interest

Scheduled emergency room visits, identified through hospital (NACRS) data and defined as “if a surgical day/night care or an organized outpatient clinic visit taking place in the emergency department has been pre-scheduled” [52], were excluded. The dependent variable was a binary variable based on occurrence of one or more non-scheduled emergency room visits or acute hospital admissions between the date of the interRAI PC assessment and death.

### Statistical analysis

Statistical analyses were performed using SAS statistical software version 9.2. The data were analysed using cross-tabulations and chi square tests for significance at the bivariate level. Using binary variables, multivariate logistic regression analyses were performed to identify potential predictors of ERVH. Logistic regression models were derived using all statistically significant variables associated with the dependent variable in the bivariate analyses (variables from Tables 1, 2 and 3). In some cases, non-significant variables were re-examined if further consideration was warranted based on the available literature. Manual backward elimination was used to develop models of ERVH, with the final model reduced to only include variables significant at the 0.05 level.

Logistic regression does not utilize information regarding the point in time during which the event (i.e., ERVH) occurred, thereby giving an ERVH which occurred near the beginning of the follow-up period from interRAI PC assessment to death the same weight in the analysis as one that occurred closer to death. Therefore, survival analysis (or time to event analysis) was also performed to identify factors that have a significant effect on the hazard rate of when ERVH occurred. Variables from Tables 1, 2 and 3 were tested using Kaplan-Meier which helped to identify the significant variables that were entered into the Cox proportional hazards regression. Survival was measured as time in days from interRAI PC assessment to the date on which an event of ERVH occurred.

## Results

Table 4 shows that half of the 764 identified clients had one or more ERVH before death (n = 399, 52.2%). Of 399 clients, 377 visited an emergency room and three quarters of these visits resulted in a hospital admission (n = 284, 75.3%). Of 399 clients, 22 were directly admitted to the hospital with no preceding emergency room visit. Among clients who had a hospital admission (n = 306), 60.1% (n = 184) died in the hospital. Median time from interRAI PC assessment to the first ERVH was 24 days (mean 43 days, SD 50.1). Over a 24-hour period, the number of emergency room visits increased from early morning to the afternoon and decreased again in the evening. In addition, clients who had at least one ERVH had a longer survival time from interRAI PC assessment date to death (mean 90 days, median 62 days), compared to clients with no ERVH (mean 55 days, median 32 days).

**Table 4:** Emergency department visits or hospitalizations by HPC home care clients

**Characteristics**		**% (n)**
**Emergency room visits or acute hospital admissions**		
*0*		47.8 (365)
*1+*		52.2 (399)
**ED Visits**		
*0*		50.6 (387)
*1*		30.9 (236)
*2*		11.8 (90)
*3+ (max of 11)*		6.7 (51)
	**Day of week presenting to ED**	
	*Monday*	17.8 (67)
	*Tuesday*	11.6 (44)
	*Wednesday*	13.5 (51)
	*Thursday*	18.0 (68)
	*Friday*	16.7 (63)
	*Saturday*	11.6 (44)
	*Sunday*	10.6 (40)
	**Time of ED visit**	
	*Midnight to 8 am*	22.0 (83)
	*8 am to 4 pm*	57.8 (218)
	*4 pm to Midnight*	20.2 (76)
	**Died in ED**	
	*No*	94.7 (357)
	*Yes*	5.3 (20)
**Admissions**		
*0*		59.9 (458)
*1*		29.2 (223)
*2*		8.5 (65)
*3+ (max of 6)*		2.3 (18)
	**Died during admission**	
	*No*	39.9 (122)
	*Yes*	60.1 (184)

### Bivariate analysis

Tables 1, 2 and 3 show variables and their relationship with ERVH. The following client profiles were associated with *reduced* rates of ERVH: predisposing characteristics (Table 1) – aged 75 years and older, wished to die at home, wished to die now, any advance care directives; enabling factors (Table 2) – caregivers were distressed and client’s cost of care above the median; and need variables (Table 3) – previous hospitalization, bladder or bowel incontinence, weight loss, swallowing difficulties, potential delirium, pressure ulcers, peripheral edema, ADLH 1+, difficulty with daily decision making, and CHESS 3+. The following client characteristics were associated with *increased* unadjusted odds of ERVH: predisposing characteristic – married; and need variable –believed physical function could improve.

### Multivariate analysis

Table 5 shows the final logistic regression model for factors associated with having one or more ERVH. The following variables were also tested again in the multivariate model although they were not significant at the bivariate level: sex, dizziness, falls, pain, dyspnea, prior pneumonia, and prior UTI. In the final model that combines predisposing, enabling, and need variables, the following variables were no longer significant: predisposing characteristics – age and wish to die now; and need variables – weight loss, pressure ulcers, peripheral edema, and prior pneumonia. Dizziness and prior UTI were significant at the multivariate level.

**Table 5:** Logistic regression model for emergency department visits or hospitalizations

**Independent variable**	**Predisposing only**		**Enabling only**		**Need only**		**All**			
							**Model 1**		**Model 2**	
	**P.E.**	**Odds ratio**	**P.E.**	**Odds ratio**	**P.E.**	**Odds ratio**	**P.E.**	**Odds ratio**	**P.E.**	**Odds ratio**
	**(S.E.)**	**(95% CI)**	**(S.E.)**	**(95% CI)**	**(S.E.)**	**(95% CI)**	**(S.E.)**	**(95% CI)**	**(S.E.)**	**(95% CI)**
**Predisposing**										
Married	0.47	1.61					0.55	1.74	0.55	1.74
	(0.15)	(1.19-2.18)					(0.17)	(1.25-2.41)	(0.17)	(1.25-2.41)
Wish to die at	−0.84	0.43					−0.61	0.54	−0.61	0.54
Home	(0.17)	(0.31-0.61)					(0.19)	(0.37-0.79)	(0.19)	(0.37-0.79)
Any advance	−0.96	0.38					−0.94	0.39	−0.93	0.39
Directives present	(0.15)	(0.28-0.52)					(0.17)	(0.27-0.55)	(0.17)	(0.28-0.55)
**Enabling**										
Caregiver			−0.59	0.55			−0.69	0.50	−0.67	0.53
Distressed			(0.20)	(0.37-0.82)			(0.23)	(0.32-0.79)	(0.23)	(0.38-0.74)
Client’s cost of			−0.50	0.61			−0.60	0.55	−0.64	0.55
Care > Median			(0.15)	(0.45-0.81)			(0.17)	(0.39-0.76)	(0.17)	(0.39-0.76)
**Need**										
Hospitalization in					−0.43	0.65	−0.55	0.57	−0.51	0.60
Last 90 days					(0.21)	(0.46-0.91)	(0.18)	(0.40-0.82)	(0.18)	(0.41-0.86)
Believes physical					0.55	1.73	0.37	1.45	0.38	1.46
Function can improve					(0.20)	(1.18-2.55)	(0.21)	(0.96-2.10)	(0.21)	(0.97-2.21)
Bladder/bowel					−0.63	0.53	−0.39	0.68	−0.46	0.63
Incontinence					(0.19)	(0.37-0.77)	(0.20)	(0.46-1.01)	(0.20)	(0.43-0.94)
Swallowing					−0.58	0.56	−0.50	0.61	−0.52	0.59
Difficulties					(0.18)	(0.39-0.80)	(0.18)	(0.41-0.89)	(0.19)	(0.40-0.86)
Dizziness					−0.33	0.72	−0.45	0.64	−0.41	0.66
					(0.17)	(0.51-1.00)	(0.18)	(0.45-0.91)	(0.18)	(0.46-0.94)
Peripheral edema					−0.31	0.74				
					(0.17)	(0.51-1.03)				
Any cognitive					−0.63	0.53	−0.63	0.53	−0.65	0.52
Impairment (incl delirium)					(0.19)	(0.37-0.77)	(0.20)	(0.36-0.79)	(0.20)	(0.35-0.77)
Prior pneumonia					0.57	1.77				
					(0.32)	(0.94-3.33)				
Prior urinary tract					0.98	2.66	0.96	2.62	0.89	2.45
Infection					(0.41)	(1.19-5.96)	(0.42)	(1.16-3.96)	(0.31)	(1.06-5.62)
ADLH 1+					−0.44	0.66	−0.34	0.71		
					(0.17)	(0.47-0.92)	(0.18)	(0.50-1.01)		
CHESS 3+					−0.39	0.65			−0.36	0.70
					(0.17)	(0.47-0.91)			(0.17)	(0.50-0.99)
C Statistic	0.69		0.67		0.58		0.71		0.77	

Note: P.E = parameter estimate, S.E. = standard error, CI = confidence interval.

In separate models, the C-statistic for predisposing variables only was 0.69, for enabling variables only was 0.58, and for need variables only was 0.71 (where 0.5 indicates chance prediction and 1 indicates perfect prediction). These statistics suggest that the predisposing characteristics are nearly as good as the need variables in predicting ERVH among HPC home care clients.

The final model was also stratified by time to death (median = 46 days) in order to examine whether proximity to death would result in a different model. In addition, the multivariate analyses were repeated for a sub-population of clients who lived for at least 60 days because although all clients had at least a one-day opportunity to make an ERVH, some clients were only in the palliative program for as short as a week (n = 62), and so it could be assumed that they may not have had the potential to make an ERVH. Both the stratified model and the model for the sub-population of clients who survived to at least 60 days showed many of the same variables as the full model, with no new significant variables in those models.

### Survival analysis

Both logistic regression and Cox regression should yield similar results when the length of the follow-up is sufficiently short. Since the length of the follow-up from interRAI PC assessment to death varied among the sample, ranging from a few days to one year, the final models were not identical; however, the models did include many of the same variables (Table 6). This finding suggests that there was a strong agreement between both models that the following variables are all important independent predictors of the likelihood of ERVH among HPC clients: predisposing characteristics – wish to die at home and any advanced care directives; enabling factor – client’s cost of care; and need variables – dizziness and prior UTI.

**Table 6:** Cox regression model building for emergency department visits or hospitalizations

**Independent variable**	**Predisposing only**		**Enabling only**		**Need only**		**All**	
	**P.E**	**Hazard rate**	**P.E**	**Hazard rate**	**P.E**	**Hazard rate**	**P.E**	**Hazard rate**
	**(S.E.)**	**(95% CI)**	**(S.E.)**	**(95% CI)**	**(S.E.)**	**(95% CI)**	**(S.E.)**	**(95% CI)**
**Predisposing**								
Female	−0.22	0.80						
	(0.10)	(0.6-0.97)						
Wish to die at home	−0.36	0.69					−0.36	0.70
	(0.13)	(0.54-0.90)					(0.14)	(0.53-0.91)
Any advance	−0.27	0.78					−0.30	0.74
Directives present	(0.11)	(0.62-0.94)					(0.11)	(0.60-0.92)
**Enabling**								
Client’s Cost of Care			−0.54	0.58			−0.51	0.60
> Median			(0.10)	(0.47-0.71)			(0.10)	(0.49-0.74)
**Need**								
Swallowing					−0.27	0.76		
Difficulties					(0.13)	(0.59-0.99)		
Dizziness					−0.24	0.79	−0.27	0.76
					(0.11)	(0.63-0.98)	(0.11)	(0.61-0.96)
Prior pneumonia					0.38	1.46		
					(0.19)	(0.98- 2.16)		
Prior urinary tract					0.48	1.63	0.71	2.04
Infection					(0.23)	(1.03-2.57)	(0.24)	(1.28-3.24)

Note: P.E = parameter estimate, S.E. = standard error, CI = confidence interval.

## Discussion

Half of the identified clients had at least one or more ERVH during the time period from interRAI PC assessment to death. Because there is variability in length of follow up among studies and there were 22 clients directly admitted to hospital, direct comparisons of emergency room use in this group of clients with that reported in literature may not always be appropriate. Other researchers have reported rates of emergency room use of 6.0% [12], 17.0% [13], 26.6% [11], and 35% [15] among palliative clients. However, those studies had varied observation periods before death (i.e., 1 year, 6 months, 3 months, 1 month). The proportion of clients with at least one ERVH is much higher in this study than in other studies due in part to the longer observation of up to one year before death. Therefore, clients may not have come to terms with the fact that they were nearing death, and so they were more willing to go to the hospital to continue aggressive treatments if they hoped that their health could improve.

Of the clients who had an emergency room visit, three quarters were admitted and more than half died in the hospital. It may be the case that clients who were in their final moments of life and who were in need of resources that can only be provided in a hospital were using emergency rooms as access for hospital admissions.

Emergency room visits occurred during daytime hours when clients had access to physicians and home care services and the proportions dropped on weekends, perhaps suggesting that the emergency room visits were connected to calls to physician offices on weekdays where the direction was given to go to the emergency room. Therefore without the physicians’ confirmation clients might otherwise wait. A previous study reported a similar finding [11]. In addition, clients with at least one ERVH had a longer survival time compared to clients without any ERVH. Perhaps the HPC provided in hospitals are associated with the longer survival period, as recent studies have indicated that HPC may be associated with prolonged life expectancy for some patient populations [53-56].

### Predisposing characteristics

The only factor that increased the likelihood of ERVH among the predisposing characteristics was marital status. Clients who were married were more likely to have an emergency visit or hospital admission. Although individuals at the end of life have multiple caregivers, more than half are cared for primarily by their spouses [57]. It is unclear how being married operates to increase the likelihood of ERVH, and additional study of this finding may help to develop testable hypotheses.

At the end of life, the quality of care is highly dependent on eliciting client and family caregiver wishes. Clients who wished to die at home and clients who had any advanced care directives present had reduced odds of ERVH. This finding is supported by Schonwetter et al. (2008) who reported that clients with a do-not-resuscitate order prefer less aggressive treatment and tend to place greater emphasis on quality of life rather than on quantity of life [13].

### Enabling factors

Among the enabling factors, caregiver feelings of distress was associated with reduced odds of ERVH. It may be possible that caregivers’ levels of distress were based on the clients’ proximity to death. Although caregivers were distressed, they might have understood and accepted that their loved ones were approaching death, and therefore wanted to keep them at home to die in a familiar environment surrounded by their family and friends.

Clients whose cost of home care was higher than the median ($562.90) had reduced probability of ERVH, suggesting that clients with higher levels of use of home care services use ERVH less often. This finding is supported by Seow et al. (2010) who reported that more hours of home care services (including nursing to alleviate pain and dyspnea before these symptoms exacerbate and personal support services to prevent caregiver burnout) were associated with reduced use of acute care [58]. When hours of services are increased, service providers are able to anticipate and address client needs at home, thereby avoiding future emergency room visits. The present analyses do not consider home care use over time, so it is not possible to determine whether changes in service levels are associated with differences in ERVH.

### Need variables

As clients approach death they enter a phase of progressive health conditions that affect one or more organ systems. Symptoms such as bladder or bowel incontinence, swallowing difficulties, and dizziness have all been associated with reduced odds of ERVH. Although these symptoms are not unusual near the end of life, they may not require invasive treatment and they may not be alarming when understood and expected [59]. Therefore, the proximity of these symptoms to expected death may explain their negative association with ERVH use if death at home is the preferred outcome.

Similarly, clients whose needs were assumed to be the greatest based on CHESS scores of 3+, indicating increased frailty and health instability, had reduced odds of ERVH. In general, clients with unstable health may be closer to death and may have adjusted psychologically and emotionally to their illnesses. Further, clients in close proximity to death might prefer increased comfort care provided through formal home care services in the community over more aggressive treatments provided in hospital settings. Since many of the assessed needs expected to drive ERVH were not predictors, other factors that contribute to ERVH among HPC home care clients require further consideration (e.g., injury, choking).

The only evaluated need variables that were found to increase the risk of ERVH were prior pneumonia and prior UTI. Cintron et al. (2003) reported that pneumonia was a significant predictor of hospital admission among clients receiving hospice care [12]. Clients could still be experiencing symptoms even after going to the hospital, if the infections were not properly treated or if the antibiotics prescribed were not effective. Such infections may also be related to the client’s general health state. For example immobility, swallowing difficulty, and dehydration are common among end of life clients and increase the risk of pneumonia and UTI [51]. Therefore, even with proper treatment, infections could still reoccur throughout the client’s last few months or weeks of life. It should be noted that some outcomes such as prior pneumonia and prior UTI could also be viewed as a proxy for health status. Therefore, it might not have been the infection, but rather the health status of the client related to the infection that led to the ERVH.

Although the majority of the population did have access to a physician, it is unclear what that access entails. Therefore, a few assumptions are made based on a study by Nagy-Agren et al. (2002). Clients with such infections may have increased odds of ERVH because physicians may decide to offer comfort measures to reduce pain rather than treat the infection [60]. Physicians’ choice not to treat an infection may be part of a palliative care plan since a serious infection may produce sedation and coma, prior to death [60]. Prescription of antibiotics may be part of a palliative care plan if the infection produces discomfort; however, this treatment plan may require emergency room visits for diagnostic tests, for intravenous lines if swallowing difficulties are present, and for potential adverse reactions to antibiotics [60]. Further, research is needed to explore whether antibiotic treatment is beneficial or not, particularly with respect to improving symptoms at the end of life. More specifically, future research should examine predictive variables to identify which clients may benefit from antibiotic treatment and to what extent.

Pain, the most commonly cited reason for ERVH [11,13,14], was not a significant predictor in this study. Since the majority of clients in this study had a diagnosis of advanced cancer and pain is among one of its most common distressing symptoms, alleviation of pain would be a primary focus of home care services for all of these clients [61].

Previous research has emphasized that perceived and evaluated need is a primary determinant of ERVH among older adults [21-32]. However, the goodness of fit statistic from the predisposing only model provided good discrimination in predicting ERVH with a C-statistic of 0.69, enabling had a C-statistic of 0.58, and need had a C-statistic of 0.71. These results suggest that the predisposing characteristics are nearly as good as the need variables in predicting ERVH, specifically among HPC home care clients. Although predisposing characteristics have only been found to have a moderate effect on of ERVH, in this palliative population they are a more proximate predictor. Perhaps predisposing characteristics are more important as clients approach death. Pot et al. (2009) studied clients in their last year of life compared to those not in their last year of life in terms of acute service use and determined that predisposing characteristics were strong determinants of acute service use among individuals closer to the end of life [62]. The clinical relevance of this finding is yet to be understood.

According to McKillip (1987), *needs* are problems of a target group that can be solved [63]. The main approach taken at the end of life involves setting goals of care (i.e., management strategies to relieve suffering) rather than seeking problem-based solutions for issues that may cause suffering [64]. Although symptoms (e.g., shortness of breath, delirium, and weight loss) are usually treated among older adults in the general population, symptom control or management rather than treatment for the underlying source of such symptoms may be warranted at the end of life. Therefore, *need* as a component of the individual determinants may not always be appropriate for a palliative population.

The CCAC from which this data were obtained does not differ in notable ways from the other CCACs in the province, therefore study findings can probably be generalized to the other 13 CCACs [65,66].

### Implications

For clinicians and policy makers, being armed with better evidence describing patterns of ERVH may assist in targeting and addressing those risk factors that are modifiable. They can also help inform the risk adjustment of system level indicators of ERVH use in these populations.

The findings of this study may have implications for care planning as they provide a context for understanding determinants of ERVH among a HPC population at home. Although marital status has been identified as a non-modifiable risk factor associated with ERVH, the predictor is of value for informing service planners as it helps identify married clients as being at higher risk for ERVH. Identification of this predictor allows service providers to adjust their care plans to include discussions with caregivers about what to expect during the client’s dying process, including physical symptoms and psychological issues that accompany it. Increased knowledge may reduce caregivers’ anxieties by helping them to anticipate issues and learn what to do under various circumstances, thus reducing unnecessary ERVH. Identification of marital status as a non-modifiable risk factor could be used for risk-adjustment when comparing outcome measures (e.g., ERVH) across CCAC sites.

Clients’ wishes to die at home and their advance care directives were protective against ERVH. This finding has implications for the interRAI PC, which asks clients about their preferences. Although documentation of client wishes is important in providing good quality care, it is difficult for clients to accurately predict their future preferences, emotions, and behaviours. Further, decisions around end of life treatment options may change over time [67-69]. Discussions around advance care directives and preferred place of death should be conducted as a process through which clients are able to reflect on their preferences after experiencing a change in their health. Hence, more than one interRAI PC assessment should be completed as clients’ health statuses change or deteriorate.

Prior pneumonia and prior UTI as modifiable risk factors have implications for education provided to service providers, caregivers, and clients. Discussions are needed to improve the identification of signs and symptoms of infections at the end of life and their documentation in the interRAI PC. Thus, anticipation and early identification will support the development of a care plan to avoid ERVH. In cases of aspiration pneumonia, where swallowing difficulty is the main risk factor, speech language pathologists help to develop compensatory strategies for swallowing efficiency, and nutritionists provide supportive dietary modifications [70].

The results of this study helped to identify aspects of the Andersen-Newman framework that could be revised to guide research concerning ERVH among HPC home care clients. The framework should emphasize knowledge or awareness of prognosis (e.g., I will only be alive for another 6 months) as it is different than knowledge of the disease (e.g., I have advanced cancer) and preferred location of death (home or hospital). Both may influence ERVH at the end of life and fit into the beliefs component of the predisposing characteristics. In addition, the framework should consider placing infections in the evaluated component of the need characteristics, separate from the symptoms and diagnoses variables.

### Limitations

Some limitations in this study should be noted.

Although documentation of client wishes is important in providing good quality care, end of life preferences and wishes are dynamic. Since clients’ emotional context at time of prediction may differ considerably from one experienced at time of the future event, decisions around end of life treatment options may change over time. Therefore, clients’ wishes to die at home and any directives present may not accurately represent clients’ actual preferences at every point near their end of life.

The main limitation of this study is the single observation for the independent variables. Only one interRAI PC assessment was completed for all clients, although survival times ranged from a few days to one year. Clients assessed closer to death are more likely to show symptoms than those further away as the disease processes advance. Some measurements are probably stable i.e. comorbid conditions, but others will change as time to death grows closer (e.g., physical dependency, symptoms, infections, and advanced care directives). Having repeat interRAI PC assessments would have allowed a more dynamic evaluation of the impact of transitions in health.

## Conclusion

This study suggests that predisposing characteristics (wish to die at home and advanced care directives) are nearly as important as need variables (CHESS 3+, prior pneumonia, and prior UTI) in predicting ERVH among HPC home care clients. These results point to the potential opportunities to reduce ERVH by managing infections, as well as the possibility that increased home care services may reduce the rate of ERVH at the end of life.

These findings point to the potential benefit of specifying the intervals at which palliative clients should be reassessed with the interRAI PC. Clients are currently assessed once and then re-assessed based on changes in their condition; however, they would benefit from being re-assessed based on the severity of their health condition at time of the first assessment. It is recommended that clients should be re-assessed every 6 months if they are determined to live more than 6 months and more frequently assessed, perhaps every few weeks, for those with a higher risk of near term mortality. Ongoing assessments may be essential in reducing ERVH as they will allow for care and service plans to be adjusted based on clients’ changing health needs and end of life preferences.

This study also showed that clients receiving more than the median cost level of home care services were less likely to have ERVH. This suggests that enhanced support for community based services may help to avoid the costs of more expensive hospital services for persons at the end of life.

## Abbreviations

ADLH: Activities of daily living hierarchy scale; CHESS: Changes in Health End stage disease and Signs and Symptoms scale; CHRIS: Client Health Related Information System; CI: Confidence interval; CIHI: Canadian Institute for Health Information; DAD: Discharge Abstract Database; ERVH: Emergency room visits or hospitalizations; HPC: Hospice palliative care; interRAI: Group of International Researchers (http://www.interrai.org); interRAI PC: Resident Assessment Instrument for Palliative Care; MOHLTC: Ministry of Health and Long Term Care; NACRS: National Ambulatory Care Reporting System; PE: Parameter Estimate; SE: Standard Error; UTI: Urinary Tract Infection.

## Competing interests

The authors declare that they have no competing interests.

## Authors’ contributions

LSW and JPH proposed the study. LSW conducted the data analysis, interpreted the data and the results, and prepared the manuscript. JWP reviewed the analysis, participated in the interpretation of the data and in the revision of the manuscript. JB participated in the discussion of the results and in the revision of the manuscript. JPH supervised the study, participated in the interpretation of the data and in the discussion of the results. All authors read and approved the manuscript. LSW takes responsibility for the manuscript.

## Authors’ information

Lialoma Salam-White. MSc. Manager, Decision Support, Hamilton Niagara Haldimand Brant Community Care Access Centre (HNHB CCAC), Ontario, Canada.

John P. Hirdes. PhD. Ontario Home Care Research and Knowledge Exchange; Professor, School of Public Health and Health Systems, University of Waterloo, Waterloo, Ontario, Canada; and Senior Canadian Fellow & Board Member, interRAI.

Jeffrey W. Poss. PhD. Associate Adjunct Professor, School of Public Health and Health Systems, University of Waterloo, Waterloo, Ontario, Canada; and Seconded Consultant, Hamilton Niagara Haldimand Brant Community Care Access Centre (HNHB CCAC), Ontario, Canada.

Jane Blums. Direction, Decision Support, Hamilton Niagara Haldimand Brant Community Care Access Centre (HNHB CCAC), Ontario, Canada.

## Pre-publication history

The pre-publication history for this paper can be accessed here:

http://www.biomedcentral.com/1472-684X/13/35/prepub
